# Chronic Diarrhea Owing to Microscopic Colitis: A Cohort Study with Insights into Diagnostic Challenges and Size of the Problem

**DOI:** 10.3390/diagnostics14202333

**Published:** 2024-10-20

**Authors:** Ahmed Ibrahim Gad, Sara Mohamed Salem, Hanaa A. Nofal, Hayam Rashed, Hossam Tharwat Ali, Noura Almadani, Rasha Mahfouz, Nevin F. Ibrahim, Ayman M. E. M. Sadek

**Affiliations:** 1 Internal Medicine Department, Faculty of Medicine, Zagazig University, Zagazig 44519, Egypt; ahmedgadmed@gmail.com (A.I.G.); sara.m.salem2030@gmail.com (S.M.S.); nova.fayek@gmail.com (N.F.I.); ayman.sadek@zu.edu.eg (A.M.E.M.S.); 2 Community, Environmental Occupational Medicine Department, Faculty of Medicine, Zagazig University, Zagazig 44519, Egypt; hanofal@medicine.zu.edu.eg; 3 Pathology Department, Faculty of Medicine, Zagazig University, Zagazig 44519, Egypt; hayam_rashed@yahoo.com; 4 Qena Faculty of Medicine, South Valley University, Qena 83621, Egypt; 5 Community and Psychiatric Mental Health Nursing Department, College of Nursing, Princess Nourah bint Abdulrahman University, Riyadh 11671, Saudi Arabia; naalmadani@pnu.edu.sa (N.A.); rmmahfouz@pnu.edu.sa (R.M.)

**Keywords:** microscopic colitis, chronic diarrhea, colonoscopy, fecal calprotectin

## Abstract

Background: Microscopic colitis (MC) is a recognized cause of chronic diarrhea and is often underestimated when a colonoscopy appears normal. This study aims to accurately diagnose chronic diarrhea through histopathological examination of colonoscopic mucosal biopsies and assess the prevalence of microscopic colitis and the diagnostic value of biomarkers. Methods: A hospital-based cohort study was conducted on 116 patients with chronic diarrhea. Colonoscopies and colonic mucosal biopsies were performed and analyzed, along with various tests including fecal calprotectin (FC) level, erythrocyte sedimentation rate (ESR), C-reactive protein (CRP), stool analysis, routine laboratory tests, and clinical data related to nocturnal diarrhea, abdominal pain, and unexplained weight loss. Results: In the study group, 32.8% had MC, with 25.9% having lymphocytic colitis (LC) and 6.9% having collagenous colitis (CC). Patients with MC had significantly higher FC, ESR, and CRP levels than those without colitis (*p* < 0.001). Factors associated with MC included nocturnal diarrhea (OR = 4.26; 95% CI [1.64–11.08]; *p*-value = 0.003) and abdominal pain (OR = 4.62; 95% CI [1.85–11.54]; *p*-value = 0.001). ESR at a cutoff >14 mm/h and FC at a cutoff >64 mcg/g showed excellent validity in diagnosing MC with area under the curve (AUC) values of 0.94 and 0.97, respectively. Conclusions: Microscopic colitis, particularly LC-type, is not an uncommon cause of chronic diarrhea, especially when accompanied by symptoms such as abdominal pain and nocturnal diarrhea, warranting further investigation, including inflammatory markers and colonic biopsy. Inflammatory markers can be useful in diagnosing MC with proper values and approaches; however, further studies are needed.

## 1. Introduction

Microscopic colitis (MC) is a chronic inflammatory condition of the large intestine, distinct from “classical” inflammatory bowel disease (IBD) by the nearly normal appearance of the colonic mucosa and characteristic histological features, including increased lymphocytic infiltrates with or without collagen deposition. MC has two major subtypes, lymphocytic colitis (LC) and collagenous colitis (CC) [1].

The incidence of MC is rising globally, particularly in women. Although autoimmune disorders like rheumatoid arthritis and thyroiditis have been reported in MC patients, definitive immunological evidence linking these conditions is lacking [2].

Clinically, MC predominantly presents with watery diarrhea, which can occur suddenly or gradually. Patients typically experience four to nine bowel movements per day, sometimes exceeding ten, often accompanied by abdominal cramps and nocturnal bowel movements. Abdominal pain is reported in approximately 47% of cases, while weight loss occurs in about 41% of patients [3].

Numerous potential biomarkers for microscopic colitis (MC) have been explored, but none have been proven to be diagnostic. Macroscopic abnormalities have been observed in about one-third of MC patients; however, these findings are non-specific. As a result, histological examination of colonic biopsies remains the only definitive method for diagnosing MC [3,4].

For lymphocytic colitis (LC), histology reveals chronic inflammatory infiltration characterized by an excess of lymphocytes and plasma cells in the lamina propria, with more than 20 lymphocytes per 100 epithelial cells. In collagenous colitis (CC), a key feature is the thickening of the subepithelial collagen layer to over 10 µm [4].

The diagnostic criteria for microscopic colitis (MC) include increased chronic inflammatory infiltration in the lamina propria, elevated intraepithelial lymphocytes (IELs), degeneration of the surface epithelium, and increased crypt mitosis. For lymphocytic colitis, more than 20 IELs per 100 intercryptal epithelial cells are required (normal is <1–5/100). In collagenous colitis, a subepithelial collagen band thickness greater than 10 μm is necessary for diagnosis [5].

The fecal calprotectin (FC) cutoff values for IBD are 50 mcg/g with a specificity of 0.60 and a sensitivity of 0.92, 100 mcg/g with a specificity of 0.66 and a sensitivity of 0.84, and 250 mcg/g with a specificity of 0.82 and a sensitivity of 0.80 [6]. No defined cutoff values currently exist for MC.

Despite being a well-known cause of chronic diarrhea, it is often overlooked in the setting of normal colonoscopic findings. Endoscopists and pathologists need to be aware of this problem for proper management. This study aims to evaluate the prevalence of MC among patients with chronic diarrhea through histopathological examination of colonoscopic mucosal biopsies and explore the diagnostic parameters in predicting MC.

## 2. Patients and Methods

### 2.1. Study Design

A hospital-based observational prospective cohort study was carried out on 116 patients with chronic diarrhea who were in the outpatient clinic or the inpatient ward of the Gastroenterology and Hepatology unit in the Internal Medicine Department, Faculty of Medicine, Zagazig University Hospitals for colonoscopy with taking colonic mucosal biopsies. Histopathological examinations were performed at the Pathology Department, Zagazig University Hospitals. The study duration extended from January 2024 to July 2024. The study was registered on ClinicalTrials.gov, identified by Code No. ID: NCT06530836.

### 2.2. Patient Selection and Data Collection

To be eligible for this study, the patient had to fulfill the following inclusion criteria: patients of either gender who were in the outpatient clinic or the inpatient ward of the Gastroenterology and Hepatology unit in the Internal Medicine Department, aged above 18 years old, and had chronic watery diarrhea, lasting for 4 or more weeks. Diarrhea is a common term used to describe loose/watery stools, which occur three or more times within 24 h. To be considered chronic, symptoms must be ongoing for four or more weeks [7]. We excluded patients with infectious colitis, inflammatory bowel diseases (such as ulcerative colitis, Crohn’s disease, and indeterminate colitis), systemic diseases associated with chronic diarrhea (e.g., diabetes mellitus and thyroid disorders), those taking medications that cause chronic diarrhea (e.g., antibiotics, antidepressants, and angiotensin-converting enzyme inhibitors, proton pump inhibitors, non-steroidal anti-inflammatory drugs), and individuals with chronic diarrhea due to malabsorption, such as pancreatic insufficiency or bile acid deficiency.

### 2.3. Clinical and Laboratory Assessments

Data was collected for each eligible patient in the study through comprehensive history-taking encompassing stool consistency, number of daily defecations, duration of diarrhea, as well as other gastrointestinal symptoms such as abdominal pain, weight loss, and prior medication, along with a thorough clinical examination. Baseline laboratory tests, including complete blood count (CBC), thyroid-stimulating hormone (TSH), fasting blood glucose (FBG), 2 h postprandial blood glucose (PPBG), electrolyte levels, liver function tests, renal function tests, and coagulation tests were conducted. Additionally, tests were performed for erythrocyte sedimentation rate (ESR), C-reactive protein (CRP), stool analysis, stool culture, and FC. Each patient underwent a total colonoscopy with terminal ileal intubation by experienced endoscopists, and colonic mucosal biopsies were microscopically examined by expert pathologists.

#### 2.3.1. Samples

Blood and stool samples were obtained in BD Vacutainer tubes (Becton, Dickinson and Company, Franklin Lakes, NJ, USA), with six tubes taken from each patient: one citrate, one ESR, two plain, and two EDTA tubes. One EDTA tube was used for a complete blood count. The plain Vacutainer tube was left to clot for 30 min, after which the sample was centrifuged at 1200× *g* for 10 min to separate the serum. The citrate tube was immediately centrifuged at 2000× *g* for 15 min for coagulation testing. Colonic mucosal biopsies were obtained from all segments of the colon, including any abnormal-looking areas, the right colon (cecum, ascending colon, and transverse colon), the left colon (descending colon and sigmoid colon), and the rectum.

#### 2.3.2. Laboratory Tests

The CBC was performed by the XS500i Hematology analyzer (Sysmex, Kobe, Japan). The differential cells were counted using the blood film. The ESR was measured by the Westergren method. Coagulation tests were performed with a Sysmex CS2100i (Siemens, Munich, Germany). All biochemical tests were quantified using the Cobas 8000 Modular Analyzer (Roche Diagnostics, Mannheim, Germany). FC concentration was determined by the Calprest ELISA (Eurospital, Trieste, Italy) according to the manufacturer’s specifications.

#### 2.3.3. Histopathology

The biopsies of all studied cases were fixed in 10% formal saline, processed routinely, and stained with hematoxylin and eosin for morphological details. Final diagnosis was made after proper correlation with the patients’ clinical endoscopic findings. These biopsies were categorized into LC and CC.

#### 2.3.4. Immunohistochemistry Protocol

Formalin-fixed, paraffin-embedded tissue blocks were sectioned into 3–5 μm slices, deparaffinized in xylene, and rehydrated through a graded series of alcohols. Antigen retrieval was performed using a 10 mM citrate buffer (pH 6.0) in a microwave for 20 min. To block endogenous peroxidase activity, the sections were treated with 3% hydrogen peroxide for 10 min. After rinsing with PBS, the slides were incubated with a polyclonal primary anti-CD3 antibody (clone A0452). The binding of the primary antibodies was detected using the Dako EnVision™ polymer detection system (Dako, Copenhagen, Denmark), with Meyer’s hematoxylin used as a counterstain.

### 2.4. Statistical Analysis

All data underwent collection, tabulation, and statistical examination utilizing SPSS 27.0 software (IBM Corp., Armonk, NY, USA). The normality of the data distribution was assessed using the Shapiro–Wilk test. Categorical data were presented as frequencies and relative percentages. The Chi-square test (χ^2^) was employed to ascertain differences between qualitative variables as appropriate. Logistic regression was performed for variables with significant differences. Quantitative data were reported as mean ± SD (Standard Deviation) and range. Parametric data was analyzed using the independent *t*-test to assess variations in quantitative variables across distinct groups. In the case of non-parametric data, median and range were utilized for description. Specifically, the Mann–Whitney U test was applied to ascertain differences between the two groups when dealing with non-parametric data. A *p*-value of <0.05 indicates significant results and <0.001 indicates highly significant results. The predictive ability of laboratory parameters was tested using area under the curve (AUC) analysis and reporting of the C-statistic (95%CI). A receiver operating characteristic (ROC) curve for each risk score was plotted, with the AUC measuring the discrimination.

## 3. Results

### 3.1. Demographic, Clinical, and Laboratory Data of the Patients

The study included 116 patients attending the Gastroenterology and Hepatology unit in the Internal Medicine Department. Table 1 shows basic demographic and clinical findings. The patients had a mean ± SD age of 47.47 ± 5.29 years, 61 (52.6%) patients were males, and 24 (20.7%) were smokers. Only 30 (25.9%) had nocturnal diarrhea, while 35 (31.2%) and 15 (12.9%) had abdominal pain and unexplained weight loss, respectively. The mean ± SD hemoglobin level was 12.24 ± 0.84 g/dL. The patients had normal creatinine levels with a mean ± SD of 0.82 ± 0.15 mg/dL. As for FC, the patients had a median (range) value of 56 (22.9–194.0). Table 2 shows the detailed laboratory parameters.

### 3.2. Microscopic Colitis (MC) and Associated Demographic and Clinical Factors

**Figure 1:**
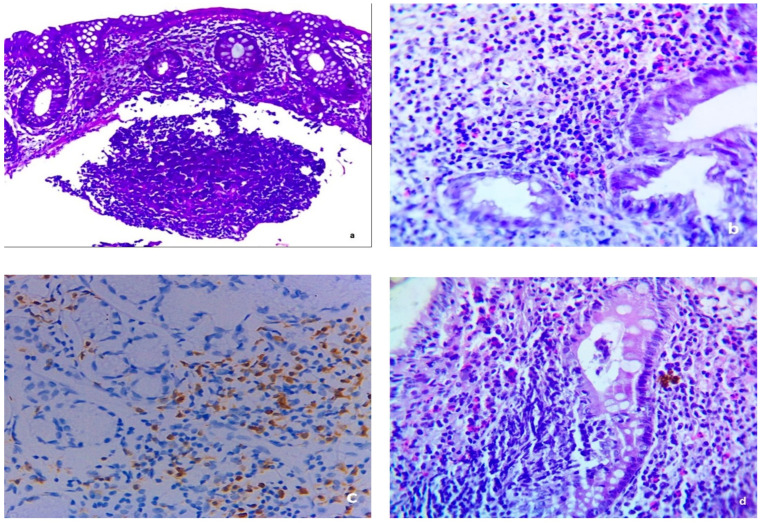
Lymphocytic type of microscopic colitis. (**a**) Sections revealed colonic intraepithelial lymphocytosis (>20 per 100 enterocytes) with a diffuse increase in lamina propria inflammatory cells (H and E × 100). (**b**) High power of the previous image (H and E × 400). (**c**) Sections revealed colonic intraepithelial lymphocytosis with a diffuse increase in lamina propria inflammatory cells stained with anti-CD3 immunostaining (IHC × 200). (**d**) The section examined revealed severe lymphocytic infiltrate and intraepithelial lymphocytosis (H and E × 400).

**Figure 2:**
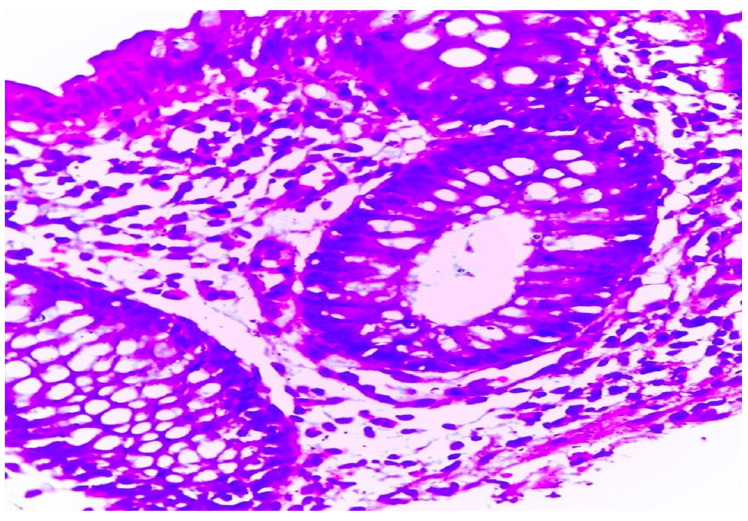
Collagenous type of microscopic colitis. Section revealed collagenous colitis with submucosal collagen deposition and lymphocytic infiltrate (H and E × 400).

In total, based on the histopathological examination, 38 (32.8%) patients were diagnosed with MC, 25.9% had LC, and 6.9% had CC. Figure 1 and Figure 2 show histopathological samples of LC and CC, respectively. Colonoscopic findings for patients were unremarkable except for four patients (3.4%) who had slight erythema, congestion, and superficial erosion. By histopathological examination, three patients (2.6%) had LC and one patient (0.8%) had CC. Regarding associated demographic and clinical factors, smoking was associated with higher risk of having MC since 50% of smokers had MS while 71.7% of non-smokers did not have MC (*p*-value = 0.04). Among the clinical findings, nocturnal diarrhea and abdominal pain were associated with greater risks of having MC; 60% of patients with each were diagnosed with MC, while 76.7% and 79% without each, respectively, did not have MC (*p*-value < 0.001 for each) (Table 3). After fitting into a binary logistic regression model, only nocturnal diarrhea (OR = 4.26; 95% CI [1.64–11.08]; *p*-value =0.003) and abdominal pain (OR = 4.62; 95% CI [1.85–11.54]; *p*-value = 0.001) were found to be associated with MC (Table 4).

### 3.3. Role of Laboratory Biomarkers with MC

**Figure 3:**
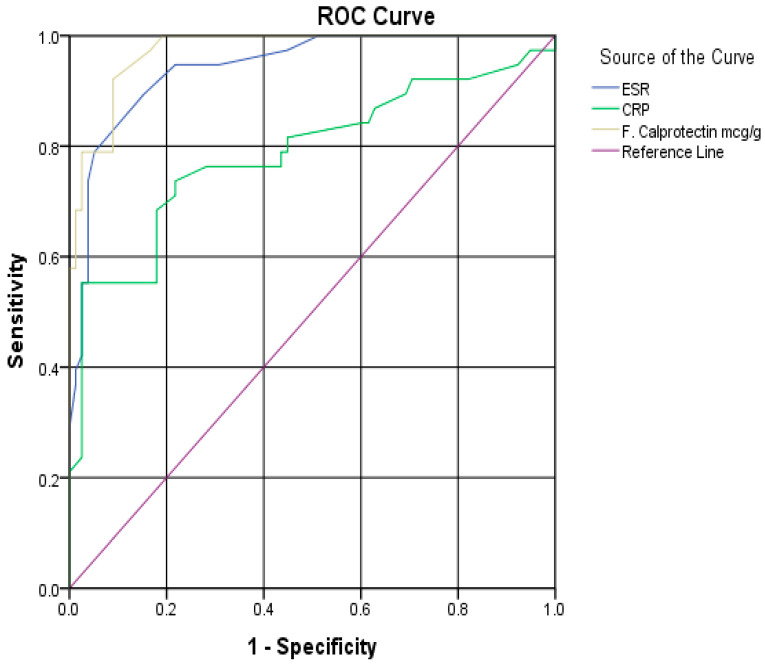
ROC curve for the performance of inflammatory markers in diagnosis of MC.

Table 5 shows that there was a statistically significant difference between the two groups regarding INR (*p*-value = 0.009). Furthermore, a highly statistically significant difference (*p*-value < 0.001) between the two groups (with MC vs. without MC) was detected in the case of median values of ESR (23 vs. 11 mm/h), CRP (7.8 vs. 4.6 mg/dL), and FC (102 vs. 42 mcg/g). Both ESR at a cutoff >14 mm/h and FC at a cutoff >64 mcg/g showed excellent performance in the diagnosis of MC with the AUC (95%CI) values of 0.94 (0.88–0.97) and 0.97 (0.92–0.99), respectively (Table 6 and Figure 3).

## 4. Discussion

Although MC is a well-known cause of chronic diarrhea, it is often underestimated in the setting of normal colonoscopic pictures. Endoscopists and pathologists need to be aware of the size of this problem for proper management. Also, despite FC having prognostic value in the assessment of inflammatory bowel disease, the relationship of FC level with MC is not determined yet. FC has evolved as a reliable fecal biomarker allowing for the detection of intestinal inflammation in IBD and infectious colitis [8].

In our study, the prevalence of MC among patients with chronic diarrhea was 32.8%, with 25.9% having lymphocytic colitis and 6.9% having collagenous colitis. A comparable prevalence was detected in a Spanish study, with 31.9% of patients with watery non-bloody diarrhea having MC [9]. Although there is a substantial variation in incidence across time and regions, most reports originate from Europe and North America [10]. The recent European guidelines reported a pooled prevalence of MC among cases with chronic watery non-bloody diarrhea of 12.8% with a significant marked heterogeneity due to geographical distributions, genetic background, definitions of MC, and workup before colonoscopy [11]. Although some studies reported greater risks with female and older patients (above 60 years), age and gender showed no significant differences between our groups [11,12]. Notably, our patients’ age ranged between 35 and 57 years.

GI symptoms, mainly nocturnal diarrhea, and abdominal pain, can be suggestive of MC as in our patients, unlike unexplained weight reduction. A previous study by Abdelmageed et al. on 100 subjects from the National Hepatology and Tropical Medicine Research Institute and Ain Shams University Hospital inpatient and outpatient clinics in Egypt concluded that distinguishing between microscopic colitis and diarrhea-predominant IBS can be facilitated by observing the presence of nocturnal diarrhea and slight weight loss. Also, collaboration between treating physicians, endoscopists, and pathologists is crucial for diagnosing MC [13].

In our study, we found that the inflammatory markers (FC, ESR, and CRP) were significantly higher in patients with MC than those without colitis. Specifically, FC and ESR showed excellent performance in predicting the diagnosis of MC. These findings can provide valuable insights into the diagnostic utility of these markers in evaluating MC. Contrary to our results, Abboud et al. revealed that ESR and CRP were not significantly correlated with disease activity in patients with MC which was predominantly of the CC type [14]. The study neither assessed FC and interleukin levels nor referred to follow-up colonic mucosal biopsy histology. Furthermore, the authors failed to specify whether the patients received steroid therapy. In our research, we specifically included patients who were newly diagnosed based on clinical manifestations and colonic mucosal histology, and who had not received any prior medication for microscopic colitis. The present study evaluates the role of CRP and ESR in predicting MC. Notably, CRP with a cut-off of 7.2 mg/dL had higher specificity among our patients (97.44%) but had an AUC of 0.78. Contrarily, the ESR with a cut-off value of 14 mm/hr had an AUC of 0.94 with a sensitivity of 89.5%. ESR and CRP are not routinely recommended for lower gastrointestinal inflammatory disorders. The American Gastrointestinal Association (AGA) recommends against the use of ESR and CRP in the diagnosis of IBD, with the use of FC or lactoferrin being preferred [15]. The present results can open future investigations into the proper use and approach of such inflammatory markers.

FC has been studied more than ESR and CRP concerning patients with diarrhea and lower gastrointestinal conditions. Although some small studies in the literature report higher FC values with MC than control, the predictive value of such parameters is low due to a large overlap [11,16]. Our results did not only show higher values with MC but also showed excellent predictive ability (AUC, 0.97) at a cut-off of 64 mcg/g with high sensitivity and specificity (92.1% and 91.03%). Another Spanish study on 94 patients with chronic diarrhea revealed the optimal cut-off for FC was >100 μg/g (AUC, 0.73), with 67% sensitivity and 75% specificity, and concluded that elevated FC concentrations are often seen in MC and may be helpful in the diagnosis of women over 60 with chronic watery diarrhea [9]. A recent systematic review and meta-analysis yielded significantly higher values of FC with MC without significant heterogeneity among studies [17].

Fecal calprotectin can be utilized in chronic diarrhea cases to differentiate inflammatory bowel diseases from functional diarrhea, such as irritable bowel syndrome. However, further research on the natural history of diarrhea as well as the optimal cut-off values is warranted to substantiate this approach. Our study depended on the diagnosis of MC after histopathology for colonic mucosal tissue that was obtained during colonoscopy. This is consistent with the study by Ashraf et al. on 172 patients with chronic watery diarrhea and normal or nonspecific colonoscopic findings. Of all the studied patients, nine cases (5.2%) had MC with six patients (3.5%) having LC and three patients (1.8%) having CC. One hundred twenty-one cases were diagnosed as chronic nonspecific colitis (70.3%). In the present study, only 3.4% patients had macroscopic findings in the colonoscopy in contrast to some previous reports of microscopic colitis [18,19]. Therefore, multiple colonic biopsies should be taken from any patient with unexplained chronic lower gastrointestinal symptoms even with a normal macroscopic picture to reach a definite diagnosis of MC [20].

The present study provides valuable insights into the available literature regarding the epidemiology, as well as the clinical and laboratory diagnosis, of MC. The strength of our study resides in the inclusion of two distinct patient groups, with and without colitis. This enables comparing FC levels, inflammatory markers, and clinical data. The present study provides the predictive abilities of ESR and CRP in MC which was not discussed in the literature. Certain limitations of our study include that the study was monocentric, inflammatory cytokines (IL-6, TNF) were not measured, and the link of FC with cytokine levels could not be assessed in the present study. Further larger studies comparing fecal and inflammatory biomarkers in patients with MS with controls and common lower gastrointestinal conditions (e.g., IBD, IBS) are recommended to confirm this study’s findings. The present study recommends further studies on the role of these biomarkers in predicting the prognosis of MC through changes during treatment course.

## 5. Conclusions

The present study indicates a high prevalence of MC among watery diarrhea cases (32.8%), with most of them having the LC subtype. Nocturnal diarrhea and abdominal pain were significantly frequent among patients with MC. Moreover, the FC, ESR, and CRP were significantly higher in patients with MC than those without colitis. Specifically, FC and ESR showed excellent predictive abilities for MC. A serial measuring of these inflammatory markers like FC, ESR, and CRP might help evaluate and monitor disease severity. Further studies are needed to validate the current findings.

## Figures and Tables

**Table 1:** Basic demographic and clinical findings of the patients.

Variable	Level	*N* (%) *N* = 116
Age: (years)	Mean ± SD Range	47.47 ± 5.29 (35–57)
Sex:	Female Male	55 (47.4) 61 (52.6)
Residence:	Rural Urban	67 (57.8) 49 (42.2)
Co-morbidities:	No HTN	99 (85.3) 17 (14.7)
Smoking:	No Yes	92 (79.3) 24 (20.7)
BMI (kg/m^2^)	Mean ± SD Range	25.26 ± 3.59 (19.8–35.2)
Nocturnal diarrhea:	No Yes	86 (74.1) 30 (25.9)
Abdominal pain:	No Yes	81 (69.8) 35 (31.2)
Unexplained weight loss:	No Yes	101 (87.1) 15 (12.9)

HTN; hypertension, BMI; body mass index.

**Table 2:** Laboratory parameters of the patients.

Variable	Level	*N* (%) *N* = 116
Hb: (g/dL)	Mean ± SD Range	12.24 ± 0.84 (10.7–15)
WBCs: (×10^3^/mm^3^)	Mean ± SD Range	9.11 ± 1.58 (4.5–13.2)
Platelets: (×10^3^/mm^3^)	Mean ± SD Range	282.06 ± 51.99 (169–374)
T. Bil.: (mg/dL)	Mean ± SD Range	0.76 ± 0.17 (0.48–1.20)
D. Bil.: (mg/dL)	Median (Range)	0.16 (0.1–0.7)
AST: (IU/L)	Mean ± SD Range	42.72 ± 11.9 (18–87)
ALT: (IU/L)	Median (range)	32.5 (14–133)
Albumin: (g/dL)	Mean ± SD Range	4.07 ± 0.30 (3.4–4.8)
TPN: (g/dL)	Mean ± SD Range	7.49 ± 0.39 (6.50–8.20)
ALP: (U/L)	Mean ± SD Range	103.22 ± 22.26 (59–153)
INR:	Mean ± SD Range	1.01 ± 0.09 (0.90–1.2)
Creatinine: (mg/dL)	Mean ± SD Range	0.82 ± 0.15 (0.38–1.2)
BUN: (mg/dL)	Mean ± SD Range	14.43 ± 2.06 (11.0–19.0)
ESR: (mm/hr)	Median (range)	13 (6–35)
CRP: (mg/dL)	Median (range)	5.6 (2–14)
FC: (mcg/g)	Median (range)	56 (22.9–194.0)

Hb, hemoglobin; WBCs, white blood cells; T. Bil., total bilirubin; D. Bil., direct bilirubin; AST, aspartate transaminase; ALT, alanine transaminase; TPN, total protein; ALP, alkaline phosphatase; INR, international normalized ratio; BUN, blood urea nitrogen; CRP, C-reactive protein; FC, fecal calprotectin.

**Table 3:** Comparison between the two groups regarding demographic and clinical data.

Variable	Level	Patients with Microscopic Colitis (*N* = 38)	Patients Without Microscopic Colitis (*N* = 78)	*p*-Value
Age: (years)	Mean ± SD Range	47.63 ± 5.3 (35.0–56.0)	47.38 ± 5.3 (36.0–57.0)	0.81
Sex:	Female (*N* = 55) Male (*N* = 61)	18 (32.7) 20 (32.8)	37 (67.3) 41 (67.2)	0.99
Residence:	Rural (*N* = 67) Urban (*N* = 49)	21 (31.3) 17 (34.7)	46 (68.7) 32 (65.3)	0.70
Co-morbidities:	No (*N* = 99) HTN (*N* = 17)	31 (31.3) 7 (41.2)	68 (68.7) 10 (58.8)	0.42
Smoking:	No (*N* = 92) Yes (*N* = 24)	26 (28.3) 12 (50.0)	66 (71.7) 12 (50.0)	0.04
BMI (kg/m^2^)	Mean ± SD Range	25 ± 2.05 (21.8–30)	25.3 ± 4.14 (19.8–35.2)	0.49
Nocturnal diarrhea:	No (*N* = 86) Yes (*N* = 30)	20 (23.3) 18 (60.0)	66 (76.7) 12 (40.0)	<0.001
Abdominal pain:	No (*N* = 81) Yes (*N* = 35)	17 (21.0) 21 (60.0)	64 (79.0) 14 (40.0)	<0.001
Unexplained weight loss:	No (*N* = 101) Yes (*N* = 15)	33 (32.7) 5 (33.3)	68 (67.3) 10 (66.7)	0.95

HTN, hypertension; BMI, body mass index. Data are represented as frequencies and percentages unless otherwise stated. Percentages are calculated per row. The *p*-value is for Chi-square and independent *t*-tests. The significance level is <0.05. Significant *p*-values are in bold.

**Table 4:** Binary logistic regression to detect variables associated with microscopic colitis.

	B	S.E.	Sig.	Odds Ratio	95% C.I.	
					Lower	Upper
Abdominal pain	1.531	0.467	0.001	4.625	1.853	11.542
Nocturnal diarrhea	1.450	0.487	0.003	4.265	1.641	11.083
Smoking	0.848	0.525	0.106	2.334	0.835	6.529

**Table 5:** Difference between the two studied groups regarding laboratory parameters.

Variable	Level	Patients with Microscopic Colitis (*N* = 38)	Patients without Microscopic Colitis (*N* = 78)	*p*-Value
Hb: (g/dL)	Mean ± SD Range	12.33 ± 0.92 (11.0–15.0)	12.2 ± 0.80 (10.7–15.0)	0.44
WBCs: (×10^3^/mm^3^)	Mean ± SD Range	9.33 ± 1.85 (4.5–13.2)	9.01 ± 1.43 (6.2–12.1)	0.34
Platelets: (×10^3^/mm^3^)	Mean ± SD Range	283.37 ± 48.84 (169.0–362.0)	281.42 ± 53.7 (169.0–374.0)	0.84
T. Bil.: (mg/dL)	Mean ± SD Range	0.74 ± 0.17 (0.48–1.1)	0.78 ± 0.17 (0.48–1.2)	0.27
D. Bil.: (mg/dL)	Median (Range)	0.15 (0.1–0.7)	0.16 (0.1–0.7)	0.35
AST: (IU/L)	Mean ± SD Range	43.97 ± 13.61 (18.0–87.0)	42.12 ± 11.16 (18.0–79.0)	0.46
ALT: (IU/L)	Median (range)	33.0 (14.0–133.0)	32.5 (14.0–102.0)	0.34
Albumin: (g/dL)	Mean ± SD Range	4.05 ± 0.31 (3.4–4.8)	4.08 ± 0.29 (3.4–4.6)	0.60
TPN: (g/dL)	Mean ± SD Range	7.55 ± 0.38 (6.8–8.2)	7.47 ± 0.39 (6.5–8.2)	0.30
ALP: (U/L)	Mean ± SD Range	107.63 ± 22.47 (59.0–153.0)	101.08 ± 21.98 (59.0–143.0)	0.14
INR:	Mean ± SD Range	0.98 ± 0.08 (0.90–1.20)	1.03 ± 0.09 (0.90–1.20)	0.009
Creatinine: (mg/dL)	Mean ± SD Range	0.81 ± 0.14 (0.38–1.10)	0.82 ± 0.15 (0.38–1.20)	0.71
BUN: (mg/dL)	Mean ± SD Range	14.43 ± 2.0 (11.0–19.0)	14.43 ± 2.09 (11.0–19.0)	0.99
ESR: (mm/hr)	Median (range)	23 (11.0–35.0)	11.0 (6.0–27.0)	<0.001
CRP: (mg/dL)	Median (range)	7.8 (2.0–14.0)	4.6 (2.2–9.4)	<0.001
FC: (mcg/g)	Median (range)	102 (58.0–194.0)	42.0 (22.9–94.4)	<0.001

Hb, hemoglobin; WBCs, white blood cells; T. Bil., total bilirubin; D. Bil., direct bilirubin; AST, aspartate transaminase; ALT, alanine transaminase; TPN, total protein; ALP, alkaline phosphatase; INR, international normalized ratio; BUN, blood urea nitrogen; CRP, C-reactive protein; FC, fecal calprotectin. The *p*-value is for independent *t*-test and Mann–Whitney U test. The significance level is <0.05. Significant *p*-values are in bold.

**Table 6:** Role of inflammatory markers in microscopic colitis diagnosis.

	Cutoff	AUC (95% CI)	Sensitivity	Specificity	PPV	NPV	Accuracy	*p*-Value
ESR (mm/hr)	>14	0.94 (0.88–0.97)	89.5%	84.6%	73.9%	94.3%	85.3%	<0.001
CRP (mg/dL)	>7.2	0.78 (0.70–0.85)	55.26%	97.44%	91.3%	81.7%	83.6%	<0.001
FC (mcg/g)	>64	0.97 (0.92–0.99)	92.1%	91.03%	83.3%	95.9%	91.3%	<0.001

AUC, area under curve; CI, confidence interval; PPV, positive predicted value; NPV, negative predicted value; CRP, C-reactive protein; ESR, erythrocyte sedimentation rate; FC, fecal calprotectin. Significant *p*-values are in bold.

## Data Availability

The data sets used and/or analyzed during the current study are presented in the results and manuscript. Further reasonable requests should be directed to the corresponding author.
